# *Nicotiana benthamiana* as a model plant host for *Xylella fastidiosa*: Control of infections by transient expression and endotherapy with a bifunctional peptide

**DOI:** 10.3389/fpls.2022.1061463

**Published:** 2022-12-01

**Authors:** Aina Baró, Pasquale Saldarelli, Maria Saponari, Emilio Montesinos, Laura Montesinos

**Affiliations:** ^1^ Institute of Food and Agricultural Technology-CIDSAV-XaRTA, University of Girona, Girona, Spain; ^2^ Institute for Sustainable Plant Protection, National Research Council (CNR), Bari, Italy

**Keywords:** bifunctional peptide, bactericidal, plant defense elicitor, plant infections, nicotiana benthamiana, *Xylella fastidiosa*

## Abstract

Transient expression of genes encoding peptides BP134 and BP178 by means of a *Potato virus X* (PVX) based-vector system, and treatment with synthetic peptides by endotherapy, were evaluated in the control of *Xylella fastidiosa* infections, in the model plant *Nicotiana benthamiana*. Transient production of BP178 significantly decreased disease severity compared to PVX and non-treated control (NTC) plants, without adverse effects. Plants treated with synthetic BP134 and BP178 showed consistently lower levels of disease than NTC plants. However, the coinfection with PVX-BP134 and *X. fastidiosa* caused detrimental effects resulting in plant death. The levels of *X. fastidiosa* in three zones sampled, upwards and downwards of the inoculation/treatment point, significantly decreased compared to the NTC plants, after the treatment with BP178, but not when BP178 was produced transiently. The effect of treatment and transient production of BP178 in the induction of defense-related genes was also studied. Synthetic BP178 applied by endotherapy induced the expression of *ERF1*, *PR1a*, *PAL*, *PALII* and *WRKY25*, while the transient expression of BP178 overexpressed the *Cath, Cyc, PR4a, 9-LOX* and *Endochitinase B* genes. Both treatments upregulated the expression of *PR1*, *PR3*, *PR4* and *CycT9299* genes compared to the NTC or PVX plants. It was concluded that the effect of BP178, either by endotherapy or by transient expression, on the control of the *X. fastidiosa* infections in *N. benthamiana*, was due in part to the induction of the plant defense system in addition to its bactericidal activity reported in previous studies. However, the protection observed when BP178 was transiently produced seems mainly mediated by the induction of plant defense, because the levels of *X. fastidiosa* were not significantly affected.

## 1 Introduction

*Xylella fastidiosa* is a Gram-negative bacterium that inhabits the xylem vessels of near 600 plants from 85 taxonomic families (EFSA, 2020). Apart from Pierce’s Disease of grapevines, which is one of the oldest known diseases caused by *X. fastidiosa* worldwide, the pathogen causes losses of economic importance in other crops, such as almond (*Prunus dulcis*), citrus (*Citrus* spp.) and olive (*Olea europaea*) (Baldi and La Porta, 2017; EPPO, 2019).

Up to now, there is no efficient cure for infected hosts (Kyrkou et al., 2018). Thus, *X. fastidiosa*-related diseases are still important threats to agriculture all over the world, mainly due to its high genetic plasticity, its xylem-limited nature, and the fact that host plants do not show symptoms during the first stages of the disease. Over the past decades, among several chemical compounds and plant extracts, antimicrobial peptides (AMPs) have been proposed as potential pesticides for disease control (Montesinos E, 2007; Jung and Kang, 2014; Breen et al., 2015). Advantages of their use include the existence of lytic peptides with potent antimicrobial and/or antibiofilm activities, defense elicitor peptides or even multifunctional peptides with reduced cytotoxicity and good biodegradability profiles (Badosa et al., 2022).

In addition, many of these AMPs have been used to create transgenic plants, some of them showing enhanced resistance to bacterial and fungal pathogens (López-García et al., 2012; Breen et al., 2015; Montesinos L. et al., 2017). The application of this technology is especially useful in controlling xylem and phloem-limited bacteria, as the expression of AMPs can be targeted into the vessel’s lumen, which is difficult to access using conventional pesticides. One example is the production of grapevine lines expressing a neutrophil elastase and cecropin B, which conferred increased tolerance and trans-graft protection to Pierce’s Disease (Dandekar et al., 2019).

Even though, as far as we know, there are no pesticides or transgenic plants based on AMPs commercialized up to date. The careful design, selection and evaluation of suitable peptides is essential for their improved development as novel and sustainable alternatives to synthetic chemical pesticides (Montesinos E. et al., 2012; Van Esse et al., 2020). BP178, a derivative peptide from the leader peptide BP100 (syn. BP134) that was designed for being expressed in plants (Badosa et al., 2013), has been successfully produced in rice seed endosperm, and conferred resistance to bacterial and fungal infections (Montesinos L. et al., 2017). Moreover, synthetic BP178 has been described as highly active against *X. fastidiosa in vitro* (Baró et al., 2020), and recently we confirmed its efficacy in controlling *X. fastidiosa* infections in almond plants (Moll et al., 2022). Synthetic BP178 was also capable of eliciting the innate immune system of tomato and almond plants (Montesinos L. et al., 2021; Moll et al., 2022). However, it is not known if the production of BP178 by transgenic host plants could protect them from *X. fastidiosa* infections.

A transient-expression system based on *Potato virus X* (PVX) has extensively been used in *N. benthamiana* plants to produce a wide range of proteins (Saitoh et al., 2001; Dickmeis et al., 2015; Röder et al., 2018), and specifically for screening AMPs against plant pathogens (Visser et al., 2012). This system has the advantage that *N. benthamiana* is a susceptible host for *X. fastidiosa* (Randall et al., 2009; EFSA, 2022), being a reliable pathosystem for testing the antimicrobial efficacy of BP178 when transiently expressed by this viral vector.

The specific aims of the present work were (i) to validate the *N. benthamiana* system as a model plant host for *X. fastidiosa*, (ii) to develop PVX plasmid constructs to produce the peptide BP178 and its parent peptide BP134 in *N. benthamiana* plants inoculated with *X. fastidiosa* IVIA 5387.2, and (iii) to study the effect of the application of the peptides by endotherapy or transient production in the population dynamics of the pathogen and in controlling infections, as well as in eliciting defense-response in the plants.

## 2 Materials and methods

### 2.1 Growth of plants and greenhouse conditions

4-week-old *N. benthamiana* plants were used for the experiments. All plants were maintained in 0.8 l pots in an environmentally controlled greenhouse at 25 ± 2 °C (day) and 18 ± 2 °C (night), with a minimum relative humidity of 60%, and with a photoperiod of 16 h light and 8 h dark. Prior to and during the experiments, plants were watered to saturation every three days, and fertilized with a 200 ppm solution of NPK (20:10:20) once a week. Also, all along the experiments, standard treatments with insecticide and acaricide were performed to avoid presence of vector insects or pests.

### 2.2 Synthesis of peptides

Peptides BP134 (KKLFKKILKYL-OH) and BP178 (KKLFKKILKYL-AGPA-GIGKFLHSAK-KDEL-OH) were synthesized (LIPPSO laboratory, Girona, Spain) using the solid phase procedure previously described (Badosa et al., 2013; Caravaca-Fuentes et al., 2021). Briefly, a Fmoc-Rink-MBHA resin (0.55 mmol/g) was used for the synthesis of BP134, and a PAC-ChemMatrix resin (0.66 mmol/g) for the synthesis of BP178. Peptides were synthesized with purity above 95% and characterized by ESI-MS (Badosa et al., 2013; Montesinos L. et al., 2021). Lyophilized peptides (acetate salts) were solubilized in sterile Milli-Q water to a stock concentration of 20 mM and filter sterilized through a 0.22 μm pore Whatman filter. Dilutions of peptides were prepared in sterile Milli-Q water to obtain the final desired concentrations.

### 2.3 PVX plasmid construction and evaluation of transient expression in plants

#### 2.3.1. pCXI BP134 and pCXI BP178 plasmid construction and *N. benthamiana* inoculation

The pCXI scaffold (Cruz et al., 1996; Lee et al., 2014; Shukla et al., 2014), containing the PVX genome sequence under the Ca35S promoter was engineered to fuse the BP134 and BP178 peptide sequences to the viral coat protein (CP) *via* the foot and mouth disease virus (FMDV) 2A sequence. Primers CX1 BP134 2A and CX1 BP178 2A (**Table 1**) were coupled with the downstream primer CX1uni to amplify the entire PVX CP from pCXI. Amplifications were carried by using 1 unit of Phusion High Fidelity DNA polymerase (ThermoFisher Scientific) in a 50 μl reaction mix containing 0.5 μM reverse and forward primers, 200 μM dNTPs, 50 ng pCXI in 1x Phusion HF buffer. Cycling conditions were: 98°C for 30” followed by 25 cycles at 98°C 30”, 54°C 30”, 72°C 45” and a final elongation step of 72°C for 10’. The obtained amplicons were digested with EagI/SpeI and ligated to a similarly restricted pCXI to obtain the plasmids pCXI BP134 and pCXI BP178 by transformation in *Escherichia coli* strain DH5α. *N. benthamiana* plants at the 6-8 leaf stage were inoculated with pCXI BP134, pCXI BP178 (two different plasmids) and pCXI by Celite abrasion of three leaves with 5 μg of purified plasmid DNA, and plants were maintained in controlled greenhouse conditions.

**Table 1:** Primer sequences used to engineer the PVX viral vector for the expression of the *BP134* and *BP178* gene peptides.

Primer code	Sequence (5’-3’)	Reference
CX1 BP134 2A	AAACGGCCGATGAGT*AAGAAACTGTTCAAGAAGATCCTTAAGTACCTC* **TCCGGA**TCTAGAAATTTTG	This study
CX1 BP178 2A	AAACGGCCGATGAGT*AAGAAGCTGTTCAAGAAGATCCTCAAGTACCTCGCTGGACCAGCTGGCATCGGCAAGTTCCTCCACTCCGCCAAG* TCCGGATCTAGAAATTTTG.	
CX1uni	GTTGTAAAACGACGGCCAGT	Lee et al., 2014

The EagI restriction site used for cloning is underlined, while the BP134 and BP178 gene peptide sequences are in italic.

Only in the case of PVX – BP134 plants, virus particles were partially purified from 100 g of *N. benthamiana* leaf tissues (Lee et al., 2014). Briefly, tissues were homogenized in 200 ml cold phosphate buffer and the slurry filtered with gauze and centrifuged at 7,800 g for 20 min at 4°C. The supernatant was added to 1% (v/v) Triton X-100, stirred for 1 h at 4°C and again clarified by centrifugation at 5,500 g for 10 min at 4°C. Virus particles, precipitated from the supernatant by NaCl/PEG stirring and centrifugation at 7,800 g for 10 min at 4°C, were resuspended in 10 ml 0.5 M phosphate buffer pH 7.2 and finally concentrated by high speed centrifugation in a swinging bucket rotor at 104,000 g for 75 min at 4°C. Pellet was resuspended in 0.2 ml of the same buffer.

#### 2.3.2. Western blot analysis of pCXI BP134 and pCXI BP178 inoculated plants

Two weeks after the inoculation, 500 mg *N. benthamiana* leaf tissues were ground in 0.5 ml of extraction buffer [EB: 50 mM Tris, 10 mM potassium acetate, 1 mM sodium EDTA, 10 mM dithiothreitol, 0.5 mM phenylmethylsulfonyl fluoride (PMSF), 10% sucrose, pH 7.4] and successively centrifuged at 4,000 rpm to separate the supernatant. Proteins were fractionated in 12% SDS-polyacrylamide gels (Laemmli, 1970) and transferred to PVDF membrane (Millipore, Bedford) (Towbin et al., 1979). Membranes were preincubated in blocking buffer [1× TBS (0.01 M Tris-HCl pH 8.0, 0.15 M NaCl), 0.05% Tween 20,5% non-fat dried milk] and probed with the antibodies raised against the PVX and the peptides (GenScript Crop, Piscataway, USA), each at the 1:1,000 dilution in the same buffer. After overnight incubation at 5°C, the membranes were washed with 1× TBS-0.05% Tween and exposed to an anti-rabbit IgG alkaline phosphatase conjugate diluted at 1:2,000. Reactions were revealed by incubation with bromo-4-chloro 3-indolyl phosphate and nitroblue tetrazolium.

### 2.4 *X. fastidiosa*

*X. fastidiosa* subsp. *fastidiosa* strain IVIA 5387.2 (ST 1) first isolated from an almond tree in Mallorca (Spain) (Moralejo et al., 2019) and reisolated in our facilities from a *N. benthamiana* infected plant, was used within the different experiments. The strain was maintained at - 80°C in Pierce’s Disease 2 (PD2) broth supplemented with 30% glycerol. When required, cryoconserved stock cultures were plated on buffered charcoal yeast extract (BCYE) solid medium and grown at 28°C for two passages of 5 days each.

### 2.5 Inoculation of *X. fastidiosa*

**Figure 1:**
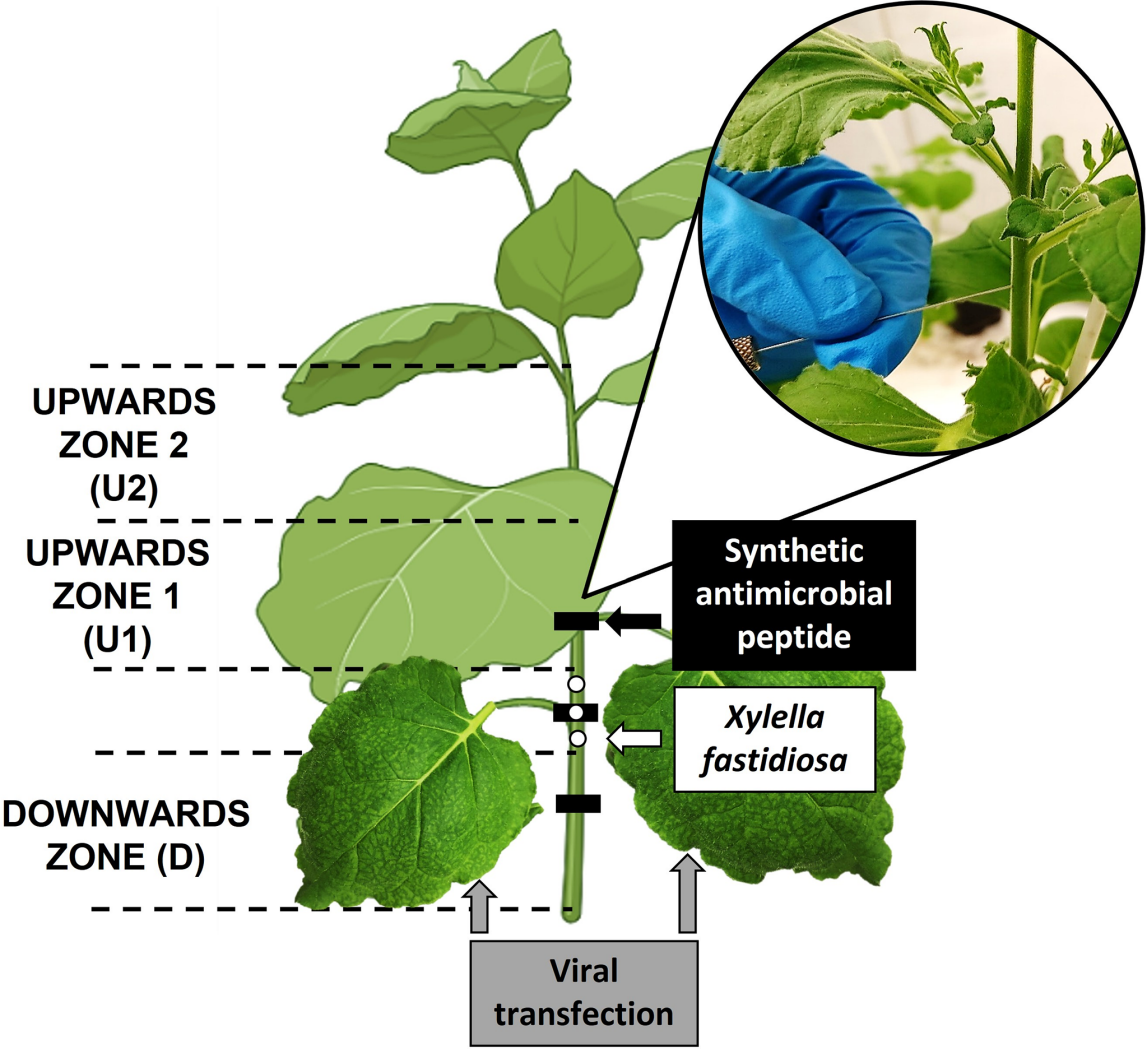
Scheme of pathogen inoculation, peptide application points, and the leaves transfected with PVX constructs, and details of inoculation/delivery process in the stem of the plant. The location of each sampling zone is also indicated: Upwards zones (U1 and U2) and downwards zone (D).

The inoculation of *X. fastidiosa* was performed by microinjection applied to the main stem as described previously (Baró et al., 2021; Moll et al., 2022). Briefly, a pathogen suspension, at 10^8^ CFU/ml (OD_600_ ≅ 0.3) confirmed by plate counting, was prepared in phosphate buffered saline (PBS), pelleted (10 min at 13,000 rpm) and resuspended in PD2 broth 1x, to avoid cell aggregation inside the syringe during injection and to ensure cell viability. Inoculations were performed using a high precision microinjector (NanoJet, Chemyx, Stafford, TX, USA) provided with a Hamilton 250 µl syringe including a thin needle with bevel tip (Bondaluz, Switzerland). The needle end was introduced into approximately one half the plant stem diameter to directly access the vascular system, as described previously. Three inoculations of *X. fastidiosa* suspension of 10 µl each (30 µl of total inoculum/plant, 3x10^6^ CFU/plant) were applied at the same side of the stem in a section of 3 cm at around 10 cm above the soil level (**Figure 1**).

Inoculated plants were cultivated in a Biosafety level II+ quarantine greenhouse authorized by the Plant Health Services, according to EPPO recommended containment conditions (EPPO, 2006), taking into consideration the quarantine status of *X. fastidiosa* in the EU (EFSA PLH Panel et al., 2018).

### 2.6 Endotherapy treatments

Endotherapy treatments with synthetic BP134 or BP178 were performed following the same procedure as described for pathogen inoculation. Briefly, three applications of 10 µl each of the stock solution at 20 mM (30 µl of peptide/plant, corresponding to 1.94 mg of BP178 or 0.84 mg of BP134 per plant) were performed at the same side of the stem in a section of 15 cm at around 5 cm above the substrate level (**Figure 1**).

### 2.7 Effect of treatments on plant infection

The effect of transient expression of *BP134* and *BP178* using the PVX system was compared with that of preventive treatments with synthetic BP134 and BP178 applied by endotherapy. In a first experiment, the effect was evaluated on the disease severity. Nine plants were used for each treatment: (i) non-treated control (NTC), (ii) synthetic BP134, (iii) synthetic BP178, (iv) PVX, (v) PVX expressing *BP134*, and (vi) PVX expressing *BP178*. Treatments, either with synthetic peptides, PVX systems or inoculations with *X. fastidiosa* suspension, were performed as described above. Preventive treatments of synthetic BP178 and BP134 were performed 24 h prior to *X. fastidiosa* inoculation, while the inoculation with PVX systems to the two selected basal leaves (**Figure 1**) was carried out one week before the inoculation of *X. fastidiosa*. Disease severity was assessed at 30 days post-inoculation of the pathogen (dpi) using the severity scale adapted from the one already described for grapevines (Clifford et al., 2013). The scale was the following 0 = no symptoms, 1 = from one to five leaves just beginning to show decay, 2 = from five to ten leaves showing significant decay, 3 = one-half or more of the leaves showing decay and few showing necrosis, 4 = all the leaves showing decay and one-half or more showing necrosis, 5 = dead plant.

In a second experiment, the effect of BP178 on the disease severity and population levels of *X. fastidiosa* was evaluated using four sets of 15 plants each: (1) NTC, (2) synthetic BP178, (3) PVX and (4) PVX expressing *BP178*. The procedure for treatments and inoculation of *X. fastidiosa* were the same as in the previous experiment, and the severity score was evaluated using the already mentioned severity scale. Three plants were assessed for disease severity and sampled for population levels at 7, 14, 21, 30 and 40 dpi. The movement and the population levels of *X. fastidiosa* in xylem tissue were determined by qPCR as described in Baró et al., 2021. Briefly, the bark was removed from the stem samples and the xylem tissue was placed in extraction bags (Bioreba AG, Switzerland) with sterile PBS (ratio 1 ml PBS/0.2 g plant tissue). Homogenization was performed using a hammer followed by the Homex 6 semi-automated homogenizer (Bioreba AG, Switzerland). Bags were incubated at 4°C for 20 min prior to obtain the pelleted cells by centrifugation of 180 µl of homogenate. GeneJET Genomic DNA Purification Kit (Thermo Fisher Scientific, USA) was used for DNA extraction. Three plants for each dpi were analyzed and upward 1 (U1, 8 cm above the inoculation point), upward 2 (U2, 8 cm above U1) and downward (D, 8 cm below the inoculation point) zones were sampled individually for each plant (**Figure 1**). The number of cells present in the stem was expressed as log_10_ CFU/g by interpolating the C_T_ values of each sample in the standard curve (y=-3.42+44.77, R^2^ 0.9997).

### 2.8 Effect of treatments on the induction of defense-related genes in plants

The expression levels of defense-related genes in *N. benthamiana* leaves in response to the treatments with synthetic BP178 and of PVX expressing *BP178* were examined. Nine plants were used for each treatment. In addition, a NTC set in which distilled water was applied by endotherapy, and a set of plants expressing empty *PVX* construct alone, were also included. Treatments with either synthetic BP178 or PVX systems were performed as described above, 24 h and 1 week before sampling, respectively. The experiment was performed twice.

Leaf samples were collected and processed to extract RNA for reverse transcription quantitative real-time PCR (RT-qPCR) assays as described in Oliveras et al., 2018. Plant material was ground to a fine powder, and total RNA was extracted from leaves using PureLink Plant RNA Reagent (Invitrogen, Life Technologies). The RNA was solubilized in RNAse free water and was routinely subjected to DNAse treatment (Ambion^®^ Turbo DNA-free™, Life Technologies). First-strand of complementary DNA (cDNA) was generated from total RNA using reverse transcriptase (High-Capacity cDNA Reverse Transcription Kit, Applied Biosystems).

To analyze gene expression in treated *N. benthamiana* plants, a quantitative real-time PCR (qPCR) was carried out in a fluorometric thermal cycler (qPCR Quant Studio 5, Applied Biosystems) by using SYBR^®^Green PCR Master Mix (Applied Biosystems) as described in Badosa et al., 2017.

Specific oligonucleotides used for the quantification of the target genes involved in plant defense mechanisms are described in **Supplementary Table 1**. For each gene system, the concentration of the primer pair was optimized to prevent non-specific reactions or artefacts that could hide the result, and the linearity within a range of number of copies of each gene was evaluated as well as the efficiency for each curve was calculated (efficiencies ranging from 80 to 98.8%, R^2^ ≥ 0.98). Melting curve analysis was performed after each amplification to verify amplification specificity. A constitutive gene (actin gene) was used as reference control.

The total reaction volume was 20 µl and the reaction mixture contained 1x SYBR^®^Green PCR Master Mix (2x), the optimized concentration of primers (either 100, 200 or 300 nM), and 2 µl of cDNA (RT reaction). The qPCR conditions were previously described by Badosa et al., 2017.

Relative quantification of gene expression was done using the ΔΔC_T_ method (Livak and Schmittgen, 2001), and the relative expression values were normalized against the actin gene as an internal control.

### 2.9 Data analysis

To test the effect of endotherapy and transient expression of *BP134* and *BP178* on the population levels of *X. fastidiosa* and disease severity in *N. benthamiana* plants, a one-way analysis of variance (ANOVA) was performed. Means were separated according to the Tukey’s test at a *P* value of ≤ 0.05. The statistical significance of the gene expression data was determined using the REST2009 Software (Pfaffl et al., 2002). Expression levels higher than 2-fold change were considered differentially expressed.

## 3 Results

### 3.1 Transient expression of peptides BP134 and BP178 with the PVX system

**Figure 2:**
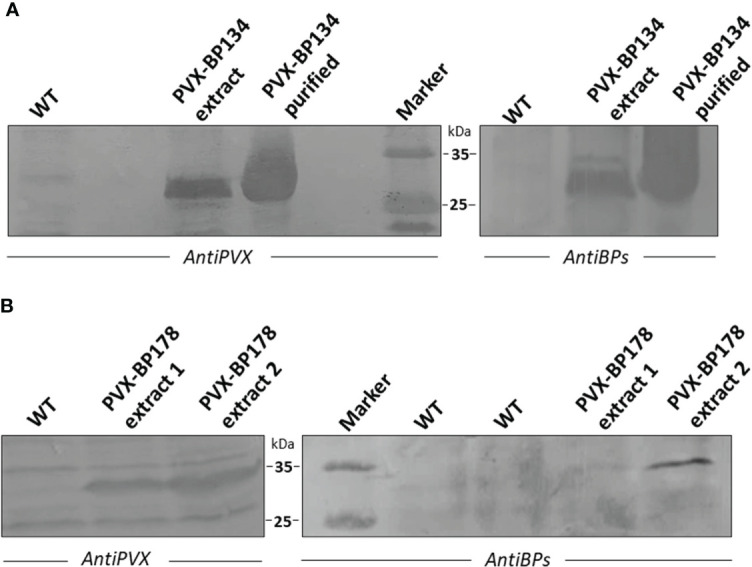
Western immunoblot analysis of PVX CP-BP134 and PVX CP-BP178 fusion proteins in total protein extracts from *Nicotiana bentamiana* leaf tissues. WT indicates protein extracts from leaves of non-transfected plants. **(A)** Extracts from PVX-BP134 and WT lines. Purified viral particles of PVX CP-BP134 were also analyzed. **(B)** Extracts from PVX-BP178 lines 1-2 and WT line. Immunodetection was performed using specific polyclonal anti-BP178/antiBP134 or antiPVX antibodies and NBT/BCIP colorimetric detection by alkaline phosphatase-conjugated antibodies.

The PVX viral vector system (Lee et al., 2014) was used to transiently express the *BP134* and *BP178* peptides in *N. benthamiana*. Engineered peptide gene sequences were fused to the viral coat protein (CP) *via* the foot and mouth disease virus (FMDV) 2A sequence, which allows the expression of free and CP-fused peptides by a ribosomal skip leading mechanism (Donnelly et al., 2001). About two weeks after the inoculation, upper leaves of plants infected with pCXI BP134 and pCXI BP178 showed mild chlorosis and deformation (not shown). Western immunoblot analysis demonstrated that both chimeric viruses correctly replicate in *N. benthamiana* (**Figure 2**), since a band recognized by the anti PVX antibody was present in transfected plants. A protein band of the same size was recognized by a mixture of anti BP134 and BP178 antibodies in the same protein extracts. In addition, anti PVX and anti BP antibodies recognized the PVX CP-BP134 fusion proteins in purified viral particles from infected plants (**Figure 2A**). Even though it is described that the FMDV 2A sequence also allows the expression of free peptides, they were not detected in any of the protein extracts.

Two different pCXI BP178 plasmids were tested, which are indicated as extract 1 and 2 in **Figure 2B**. Since the PVX CP-BP178 protein was barely visible in one of them, it was decided to further continue the experiments using the plasmid expressing the highest amount of fusion protein (**Figure 2B**). No reactions were visible in protein extracts from wild-type plants (WT).

### 3.2 Symptoms development

**Figure 3:**
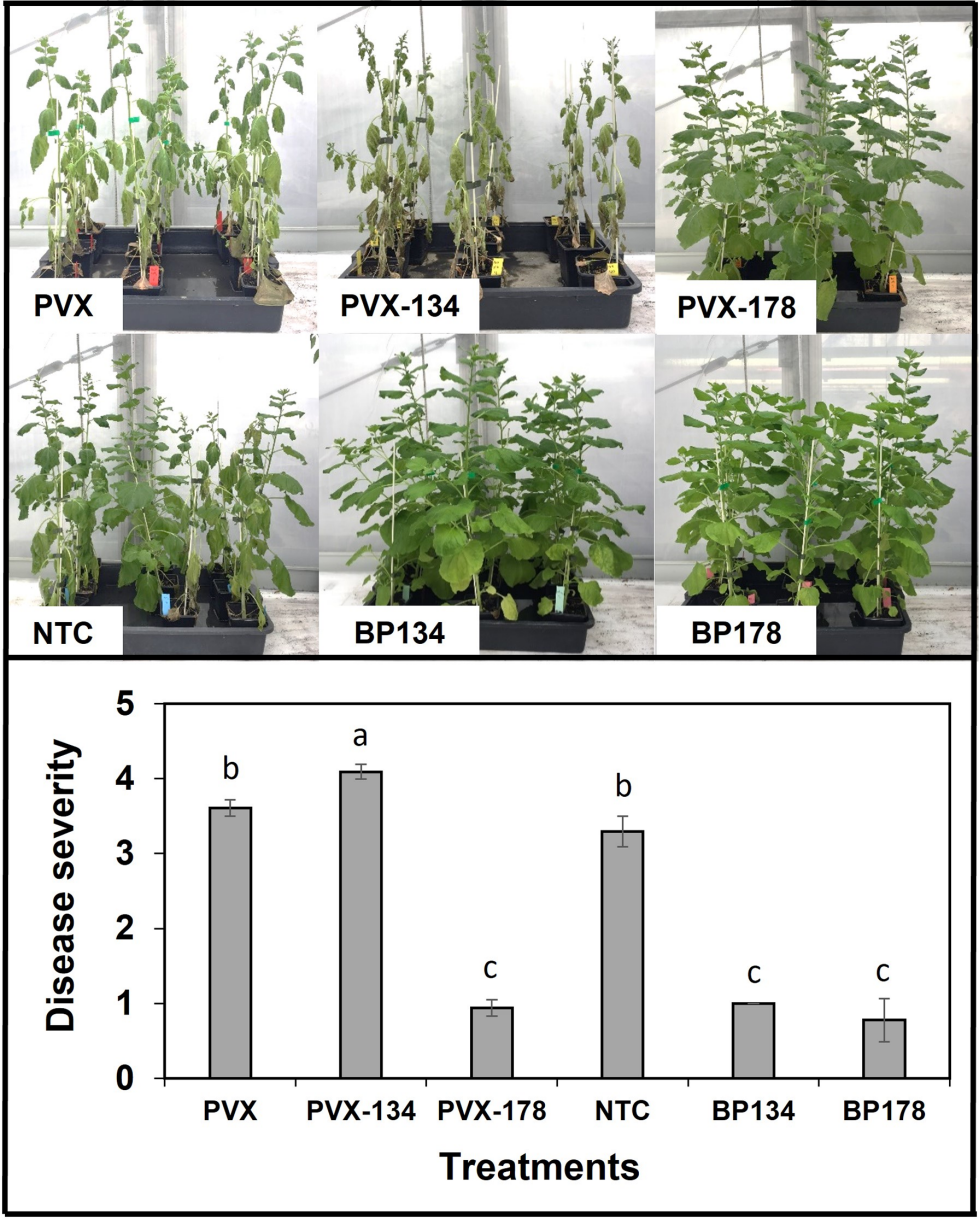
Effect of endotherapy with BP178 or BP134, and by heterologous production of the peptides on *Xylella fastidiosa* infections in *Nicotiana benthamiana* plants. The treatments were: PVX, empty vector; PVX-134, vector with BP134 gene; PVX-178, vector with BP178 gene; BP134, synthetic peptide; BP178, synthetic peptide; and NTC, non-treated control. Values are the means of nine plants (three replicates of three plants per each treatment), and error bars represent the standard deviation of the mean. Different letters between treatments correspond to statistically significant differences between treatments (Tukey’s test, *p* ≤ 0.05).

The effect of preventive treatments with the synthetic peptides, as well as their transient expression using the PVX system was assessed on the disease severity caused by *X. fastidiosa* IVIA 5387.2 in *N. benthamiana* plants at 30 dpi. Both synthetic peptides, BP134 and BP178, as well as the transient expression of *BP178* significantly decreased disease severity compared to the NTC and PVX control plants, with an efficacy of 69.7%, 76.4% and 73.8%, respectively (**Figure 3**). NTC and PVX plants showed high levels of disease, and no significant differences in disease severity were observed between them. However, the highest disease severity was observed in plants transiently expressing the peptide *BP134*. In a second transfection experiment, the preventive treatment with BP178 and the production of PVX CP-BP178 in *N. benthamiana* plants confirmed the previously observed efficacy in controlling *X. fastidosa* infections.

### 3.3 Dynamics of *X. fastidiosa*

**Figure 4:**
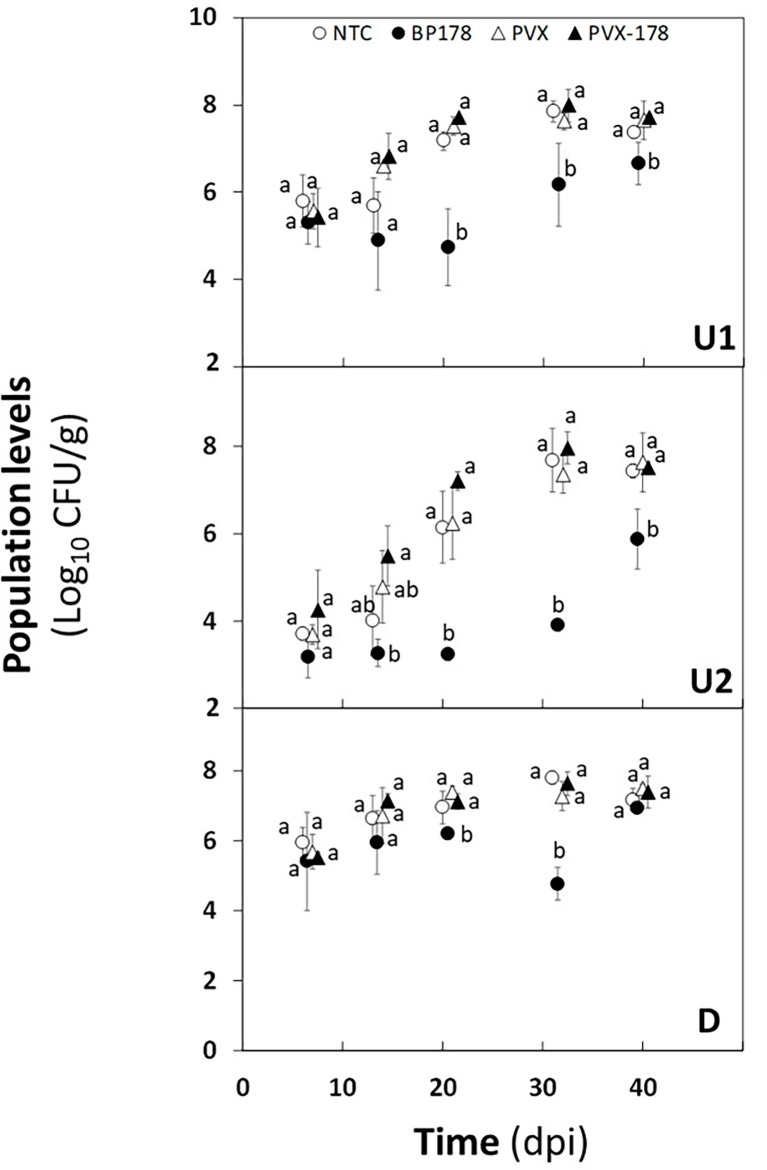
Effect of peptide treatment on *Xylella fastidiosa* subsp. *fastidiosa* population levels of *Nicotiana benthamiana* plants over a period of 40 days after pathogen inoculation. From top to bottom graph, *X. fastidiosa* population levels were estimated in the upward zones 1 (U1) and 2 (U2) and in the downward zone (D). Treatments correspond to non-treated control plants (white circle); endotherapy with BP178 (black circle); PVX transfection (white triangle); PVX-BP178 transfection (black triangle). Error bars represent the standard deviation of the mean. Different letters between treatments at specific sampling zones denote statistically significant differences between *X. fastidiosa* population levels (Tukey’s test, *p* ≤ 0.05).

The population of *X. fastidiosa* IVIA 5387.2 in *N. benthamiana* plants preventively treated or transiently expressing *BP178* is presented in **Figure 4**.

The preventive treatment with the synthetic peptide was the most effective in reducing *X. fastidiosa* cell number, with a maximum decrease of 2.4 to 3 log in all zones analyzed, compared to the NTC. Specifically, a reduction was observed in the U1 zone from 7 to 40 dpi, compared to NTC plants, and being maximum at 21 dpi. In the U2 zone, *X. fastidiosa* cells were not detected until 30 dpi (detection limit 10^3^ CFU/g). After this time point an increase in the *X. fastidiosa* population levels was observed in the U2 zone, but they were significantly lower in *BP178* plants than those in the control plants. In the D zone, even though a slight reduction was observed compared to the other treatments, the difference was not as significant as in upward zones, except at 30 dpi, where a clear decrease in population levels is observed, with a reduction of 3 logs.

There was no significant difference in the dynamics of the *X. fastidiosa* population between NTC, PVX and PVX- *BP178* plants, which increased from 7 to 30 dpi and remained stable until 40 dpi in all plant parts analyzed.

### 3.4 Induction of plant-defense related genes

**Figure 5:**
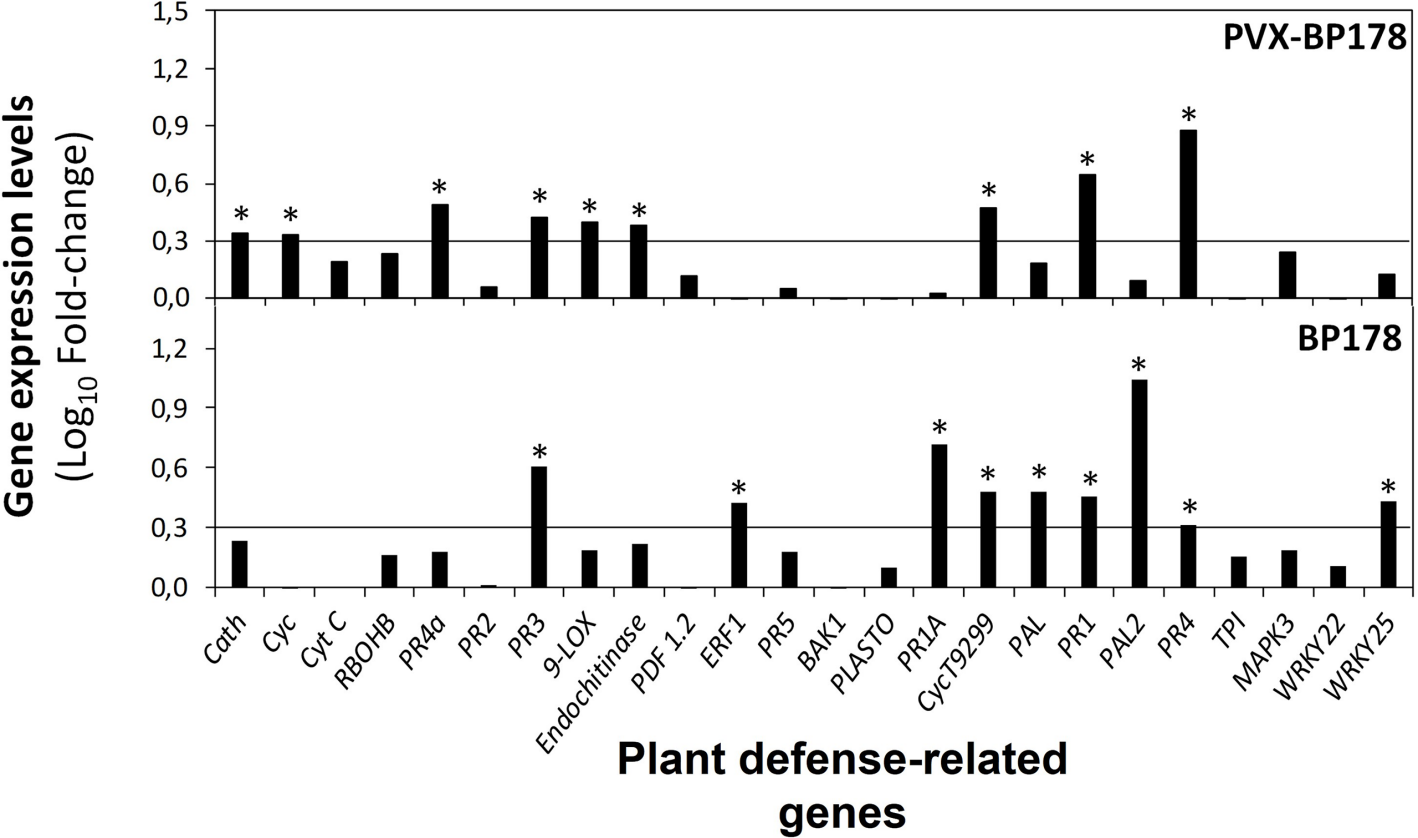
Expression levels of defense genes in *Nicotiana benthamiana* plants in response to endotherapy with synthetic BP178 or transient expression with PVX-BP178, using RT-qPCR analysis. Black line, cut-off values for genes with > 2-fold change value (log_10_, 0.3) (Relative quantification method using ΔΔC_T_ method). Each BP178 treatment was compared to its appropriate control (i.e., PVX-BP178 compared to PVX control; BP178 applied by endotherapy compared to non-treated control plants, where plants were treated with water by endotherapy). Asterisks indicate significant values of fold-change.

The ability of BP178 to induce the expression of defense-related genes was assessed in *N. benthamiana* plants using both strategies, endotherapy and transient expression. The expression of 24 genes is presented in **Figure 5** by comparison of the levels of expression when applied by endotherapy (BP178 synthetic) or transiently expressed (PVX-BP178). Both treatments significantly upregulated the expression of *PR1*, *PR3*, *PR4* and *CycT9299* genes. When transiently expressed, *Cath, Cyc, PR4a, 9-LOX* and *Endochitinase B*, genes were also upregulated. Synthetic BP178 also induced the expression of *ERF1*, *PR1a*, *PAL* and *PALII* and *WRKY25*.

The effect of PVX and PVX-BP178 compared to the NTC plants is shown in **Supplementary Figure 1**. In both, PVX and PVX-BP178 treatments, *PR2, PR3, PR5, plastocyanin, PR1a* and *PR1* genes were upregulated, while genes *Cyt C, PR4a, Endochitinase B* and *PR4* were only upregulated when *BP178* was transiently expressed.

## 4 Discussion

BP134 and BP178 peptides have potent antibacterial activity against several bacterial plant pathogens, including *X. fastidiosa* (Badosa et al., 2013; Baró et al., 2020), and are very effective in protecting plants of agronomic interests from infections caused by bacterial and fungal plant pathogens (Badosa et al., 2017; Montesinos L. et al., 2021). Recently we confirmed that BP178 applied by endotherapy and spray to almond plants was able to control infections caused by *X. fastidosa* (Moll et al., 2022). In the present work, we validated *N. benthamiana* as a model plant host for the study of *X. fastidiosa* and evaluated the effect of the transient expression of *BP134* and *BP178* in controlling infections in this model plant compared to the preventive application of the synthetic AMPs by endotherapy.

We demonstrated that *N. benthamiana* plants produce BP134 and BP178 when transiently expressed using a PVX-derived vector system. This agrees with the reports of stable genetic transformation and expression of *BP178* in rice using endosperm specific promoters (Montesinos L. et al., 2017). On the contrary, constitutive expression of *BP134* in rice plants had strong negative impact on rice fitness (Nadal et al., 2012). No yield quantification was performed in our study because when peptides are expressed using a virus-based vector system, transient production levels of heterologous peptides differ between plants, as it depends on physical factors influencing the viral vector infection, environmental conditions, and the physiological state of each plant (Sindarovska and Kuchuk, 2021). In the present study, absence of self-cleavage of the FMDV 2A sequence to obtain the free AMPs was also observed (Kovalskaya et al., 2011), which can be due to the amino acid sequence, as the ratio of fused CP and non-fused viral CP depends on it (Dickmeis et al., 2015). Even so, BP178 fused to the viral CP seems to maintain its activity when transiently produced in *N. benthamiana*, as it reduced *X. fastidiosa* disease severity with an efficacy of 74%. This observed activity agrees with the fact that BP178 has already been produced in the endosperm of rice seeds, showing a protective effect against the bacteria *Dickeya* sp. and the fungus *Fusarium verticillioides* (Montesinos L. et al., 2017).

Contrarily, PVX-BP134 transfected plants and inoculated with *X. fastidiosa* showed the highest severity index among all treatments, although the heterologous production of BP134 in non-pathogen inoculated *N. benthamiana* plants had no detrimental effect. We expected that the large fusion protein (CP-BP134) should not present toxicity because of the large size of the coat protein compared to BP134 (11 amino acids), as it was reported when the *BP134* gene alone was constitutively expressed in rice (Nadal et al., 2012). A possible explanation could be related to the effect of the coinfection itself. PVX infection leads to the synthesis of VSR HcPro in *Nicotiana tabacum*, a viral protein that suppress the plant PAMP Triggered Immunity (PTI), facilitating the multiplication of the pathogen (Navarro et al., 2008; Zvereva et al., 2016). Also, coinfection of rice with Rice yellow mottle virus and *Xanthomonas arboricola* pv. *oryzicola*, was reported to increase bacterial specific symptoms and bacterial load, compared to the bacterial infection alone (Tollenaere et al., 2017). Then, suppression of PTI by PVX together with the detrimental effects of coinfection could explain our results of an increase of symptoms during coinfection of the PVX and *X. fastidiosa*, compared to the *X. fastidiosa* infection alone. In addition, the coinfection of the PVX-BP134 and *X. fastidiosa* increased even more the disease symptoms observed with PVX and *X. fastidosa* coinfection. This should be considered together with the fact that, contrary to BP178, peptide BP134 did not have a significant induction of the plant immune system (Oliveras et al., 2018). In addition, we cannot discard that the synthesis of CP-BP134 fusion may induce tylose overproduction or other gene products that promoted and exacerbated the symptoms of the disease, but we have not analyzed tylose formation and transcriptomic profiling of PVX-BP134 plants.

Endotherapy with BP134 or BP178 reduced *X. fastidiosa* infections in *N. benthamiana* with efficacies of at least 70%, the same order as compounds used in the field to control diseases caused by this pathogen. For example, *N*-acetyl cysteine (NAC) applications reduced *X. fastidiosa* symptoms between 75 and 80% in infected sweet orange plants under greenhouse conditions (Muranaka et al., 2013), and in olive orchards with initial *X. fastidiosa* incidence, but without showing a decrease in the pathogen population levels by qPCR (Dongiovanni et al., 2017). Endotherapy treatments using oxytetracycline in infected almond trees also showed a significant reduction of disease symptoms, around 73%, related with a low infectivity percentage in leaves detected by DAS-ELISA (Amanifar et al., 2016). In terms of reduction of the levels of *X. fastidiosa*, the microinjection of BP178 was effective compared to NTC plants. This agrees with the *in vitro* bactericidal activity already reported for this functional peptide (Baró et al., 2020). Nevertheless, one single preventive application of the BP178 was not enough to prevent disease in *N. benthamiana* plants, as remaining live cells were still able to grow after 20 dpi. This agrees with the results obtained in almond plants, where increasing the number of AMP applications resulted in a higher reduction of the bacterial population and, consequently of disease severity (Moll et al., 2022).

In contrast, when BP178 was transiently produced, the population levels of *X. fastidiosa* did not decrease compared to the NTC plants, despite the observed reduction of disease severity. This was associated with a higher tolerance of these plants to the pathogen due to an elicitation of the plant immune system triggered by the production of BP178 peptide, with no direct action against the pathogen, due to the upregulation of *PR1*, *PR3*, *PR4*, *PR4a*, *CycT9299*, *Cath*, *Cyc*, *9-LOX* and *Endochitinase*.

It has been reported that tolerance to plant pathogens does not necessarily affect its growth and virulence on the host (Pagan and Garcia-Arenal, 2020). For example, salicylic acid (SA) and ethylene mediate symptom development in *Arabidopsis* plants infected with *Xanthomonas* and *Pseudomonas*, without affecting the growth of both bacterial plant pathogens (Bent et al., 1992; O’Donnell et al., 2001).

We reported that genes coding endochitinase B, PR4 and PR1 proteins were induced in *N. benthamiana* PVX-BP178 plants. However, the endochitinase B and PR4 gene products do not act directly on bacteria because they are antifungal, as confirmed in many plant species including *N. tabacum*, and have been involved in abiotic stresses such as cold, salinity or wound stress (Ali et al., 2018). In addition, PR1 has been involved in plant growth and development (Breen et al., 2017). Then, none of these gene products are expected to influence the growth of *X. fastidiosa.* Among the upregulated genes in *N. benthamiana*, we observed two cyclophilin genes, known for their role in growth and development of plants, hormone signaling (i.e., auxin signaling) and drought tolerance, but not directly related to pathogen multiplication (Singh et al., 2020). Also, cathepsin B was induced, and in the case of *N. benthamiana* it was reported to be required for disease resistance to bacterial pathogens, and for induction of SA-related genes (van der Linde, 2012a, b). In fact, the induction of *cathepsin* gene is required for the induction of *9-LOX* gene to promote cell death (Christensen et al., 2015), which also is being overexpressed in our PVX-BP178 plants. It should be considered that here we have paid attention to only 24 genes in *N. benthamiana*, but many other genes may be implicated in the plant response. Upon treatment with BP178 in tomato (microarray) or almond (RNA seq) we have identified around 100 upregulated/downregulated genes (Montesinos et al., 2021; Moll et al., 2022). Unfortunately, these extensive transcriptomic studies have not been done with PVX-BP178 *N. benthamiana* plants.

Considering our results and the possible role of tyloses in limiting pathogen progression, the overproduction of tyloses is not compatible with the spread of *X. fastidosa* we have observed within the PVX-BP178 plants. Interestingly, Ingel et al. (2020) reported that tylose formation in xylem of grapevine affected by Pierce's Disease is related to upregulation of genes associated to ethylene-signaling, cell wall biogenesis and drought stress, whereas several genes related to photosynthesis and carbon fixation were downregulated. In our case, ERF1 (ethylene transcription factor) and Cyt C (when compared to PVX) that are involved in the ethylene pathway, were not overexpressed in PVX-BP178. Unfortunately, we have not analyzed the tylose formation, and we cannot discard an effect of the peptide treatment on its formation.

Regarding the effect of BP178 in other plants like almond or tomato, *MYB transcription factor*s, osmotin, zinc fingers, terpens or PAL genes were upregulated, whereas in Pierce’s diseased grapevine plants, these genes were downregulated (Ingel et al., 2020).

Globally, we consider that transient expression of BP178 could have specific effects in the transcriptome of *N. benthamiana* that somehow compensates the negative effect of *X. fastidiosa* in host plants, but without affecting its population levels.

Since PVX is a plant pathogen, mainly affecting *Solanaceae* (Lico et al., 2015), inoculation with PVX alone induced the expression of several plant defense genes (*PR1*, *PR1a*, *PR2*, *PR3*, *PR5* and *plastocyanin*), compared to NTC plants. However, this effect was unable to prevent infections caused by *X. fastidiosa*. On the contrary, practically no symptoms were observed in PVX-BP178-infected plants in which, in addition to the induction of the genes induced by PVX alone, more genes like *Endochitinase B, Cyt C*, and two *PR4* genes, were overexpressed when comparing to NTC plants. The mechanism by which PVX promote this effect is still not well known. Virus presence in the xylem is poorly investigated although PVX was detected in the vessels of infected *N. benthamiana* (Betti et al., 2012). However, the low number of viable cells in the xylem which is mainly limited to parenchyma associated cells, makes PVX replication and AMP release in the xylem not quantitative, which could explain the lack of decrease of *X. fastidiosa* levels in PVX-*BP178*-infected plants.

Activation of the plant immune system is a strategy that has reported promising results in the control of *X. fastidiosa*. For example, the application of menadione, a plant defense activator, reduced Pierce´s Disease symptoms between 40 and 50%, (Zhang et al., 2019). Also, the foliar and drench application of abscisic acid to potted vines significantly reduced Pierce´s Disease severity compared to control plants, but only when applied in the fall (Meyer and Kirkpatrick, 2011). So, the capacity of AMPs to activate the plant immune system in addition to their antimicrobial activity, and their possibility to be successfully expressed into the lumen of xylem vessels (Agüero et al., 2005; Dandekar et al., 2012; Dandekar et al., 2019), make them of high interest for the control of systemic plant diseases.

Several plant models have been used to study *X. fastidiosa*. After the first report of *X. fastidiosa* in *Catharanthus roseus* (Ueno et al., 1998), its potential as a plant model was studied (Monteiro et al., 2001). The first symptoms appeared on some of the plants two months after inoculation with *X. fastidosa* subsp. *pauca* 9a5c, and in all the plants after four months. Similar times to symptom development were observed when *Medicago sativa* was inoculated with *X. fastidosa* subsp. *pauca* DeDonno (Kubaa et al., 2019), but in this case limited systemic colonization was observed and a low number of non-inoculated shoots showed presence of the pathogen. Similarly, some ecotypes *of A. thaliana* have been used as a plant model, with colonization of distal tissues such as floral shoots and roots and population levels around 10^6^ CFU/g already at 7 days post-inoculation (Pereira et al., 2019). However, no typical symptoms of necrosis or leaf scorch have been observed using this model plant (Rogers, 2012). *Nicotiana tabacum* was also described as a potential model (Lopes et al., 2000) and showed the first symptoms of leaf scorch between 6-8 weeks after inoculation and population levels of *X. fastidiosa* from 10^4^ to 10^8^ CFU/g tissue, but this system appears to be difficult to reproduce (Lopes et al., 2000; Alves et al., 2008; Francis et al., 2008; De La Fuente et al., 2013).

In our study with *N. benthamiana*, the strain IVIA 5387.2 causing ALS in Mallorca, showed high population levels of 10^8^ CFU/g tissue at 3-4 weeks post-inoculation in the three plant zones analyzed and, in all plants, with a rapid development of symptoms and a low variability. Thus, *N. benthamiana* is proposed as a suitable model for the study of efficacy of compounds against *X. fastidiosa* and for other studies, with advantages over the previously used plant models.

The present study gives new insights into the potential of AMPs to control diseases caused by *X. fastidiosa*, more specifically using the expression of *BP178* in plant hosts. It also emphasizes the importance of the plant model *N. benthamiana* to perform *in planta* screening of functional peptides (and eventually other compounds) against *X. fastidiosa.*

## Data availability statement

The original contributions presented in the study are included in the article/**Supplementary Material**, further inquiries can be directed to the corresponding author.

## Author contributions

EM, PS and MS obtained the financial support. AB, PS, LM, MS, and EM designed the research, analyzed the data, and wrote the paper. AB, PS and LM conducted and performed the experiments. All authors contributed to the article and approved the submitted version.

## Funding

This work was supported by grants from the European Union XF-ACTORS (Ref. 727987) and BeXyL (Ref. 101060593) projects. AB was recipient of a research grant from the Secretaria d’Universitats i Recerca, Departament d’Economia i Coneixement, Generalitat de Catalunya (Ref. 2018 FI B00334).

## Acknowledgments

We thank Héctor Saravia and Gemma Roselló for participating in various tasks related to preparation and maintenance of the plant material. We also thank Professor Ulrich Commandeur and Dr Juliane Röder from RWTH Aachen University for their precious advices about the PVX skipping mechanism.

## Conflict of interest

The authors declare that the research was conducted in the absence of any commercial or financial relationships that could be construed as a potential conflict of interest.

## Publisher’s note

All claims expressed in this article are solely those of the authors and do not necessarily represent those of their affiliated organizations, or those of the publisher, the editors and the reviewers. Any product that may be evaluated in this article, or claim that may be made by its manufacturer, is not guaranteed or endorsed by the publisher.

## References

[B1] Agüero C. B. Uratsu S. L. Greve C. Powell A. L. Labavitch J. M. Meredith C. P. (2005). Evaluation of tolerance to pierce's disease and botrytis in transgenic plants of *Vitis vinifera* l. expressing the pear *PGIP* gene. Mol. Plant Pathol. 6, 43–51. doi: 10.1111/j.1364-3703.2004.00262.x 20565637

[B2] Ali S. Ganai B. A. Kamili A. N. Bhat A. A. Mir Z. A. Bhat . (2018). Pathogenesis-related proteins and peptides as promising tools for engineering plants with multiple stress tolerance. Microbiol. Res. 212-213, 29–37. doi: 10.1016/j.micres.2018.04.008 29853166

[B3] Alves E. Kitajima E. W. Leite B. (2008). Interaction of *Xylella fastidiosa* with different cultivars of *Nicotiana tabacum*: a comparison of colonization patterns. J. Phytopathol. 151, 500–506. doi: 10.1046/j.1439-0434.2003.00759.x

[B4] Amanifar N. Taghavi M. Salehi M. (2016). *Xylella fastidiosa* from almond in Iran: overwinter recovery and effects of antibiotics. Phytopathol. Mediterr. 55, 337–345. doi: 10.14601/Phytopathol_Mediterr-17682

[B5] Badosa E. Moiset G. Montesinos L. Talleda M. Bardají E. Feliu L. . (2013). Derivatives of the antimicrobial peptide BP100 for expression in plant systems. PloS One 8, e85515. doi: 10.1371/journal.pone.0085515 24376887 PMC3871672

[B6] Badosa E. Montesinos L. Camó C. Ruz L. Cabrefiga J. Francés J. . (2017). Control of fire blight infections with synthetic peptides that elicit plant defense responses. J. Plant Pathol. 99, 65–73. doi: 10.4454/jpp.v99i0.3915.

[B7] Badosa E. Planas M. Feliu L. Montesinos L. Bonaterra A. Montesinos E. (2022). Synthetic peptides against plant pathogenic bacteria. Microorganisms 10, 1784. doi: 10.3390/microorganisms10091784 36144386 PMC9504393

[B8] Baldi P. La Porta N. (2017). *Xylella fastidiosa*: host range and advance in molecular identification techniques. Front. Plant Sci. 8. doi: 10.3389/fpls.2017.00944 PMC5462928 28642764

[B9] Baró A. Montesinos L. Badosa E. Montesinos E. (2021). Aggressiveness of Spanish isolates of *Xylella fastidiosa* to almond plants of different cultivars under greenhouse conditions. Phytopathology 111, 1994–2001. doi: 10.1094/PHYTO-02-21-0049-R 33749331

[B10] Baró A. Mora I. Montesinos L. Montesinos E. (2020). Differential susceptibility of *Xylella fastidiosa* strains to synthetic bactericidal peptides. Phytopathology 110, 1018–1026. doi: 10.1094/PHYTO-12-19-0477-R 31985337

[B11] Bent A. F. Innes R. W. Ecker J. R. Staskawicz B. J. (1992). Disease development in ethylene-insensitive *Arabidopsis thaliana* infected with virulent and avirulent *Pseudomonas* and *Xanthomonas* pathogens. Mol. Plant Microbe Interact. 5, 372–378. doi: 10.1094/mpmi-5-372 1472714

[B12] Betti C. Lico C. Maffi D. D’Angeli S. Altamura M. M. Benvenuto E. . (2012). *Potato virus x* movement in *Nicotiana benthamiana*: New details revealed by chimeric coat protein variants. Mol. Plant Pathol. 13, 198–203. doi: 10.1111/j.1364-3703.2011.00739.x 21851552 PMC6638808

[B13] Breen S. Solomon P. S. Bedon F. Vincent D. (2015). Surveying the potential of secreted antimicrobial peptides to enhance plant disease resistance. Front. Plant Sci. 6. doi: 10.3389/fpls.2015.00900 PMC4621407 26579150

[B14] Breen S. Williams S. J. Outram M. Kobe B. Solomon P. S. (2017). Emerging insights into the functions of pathogenesis-related protein 1. Trends Plant Sci. 22, 871–879. doi: 10.1016/j.tplants.2017.06.013 28743380

[B15] Caravaca-Fuentes P. Camó C. Oliveras À. Baró A. Francés J. Badosa E. . (2021). A bifunctional peptide conjugate that controls infections of *Erwinia amylovora* in pear plants. Molecules 26, 3426. doi: 10.3390/molecules26113426 34198776 PMC8201157

[B16] Christensen S. A. Huffaker A. Kaplan F. Sims J. Ziemann S. Doehlemann G. . (2015). Maize death acids, 9-lipoxygenase-derived cyclopente(a)nones, display activity as cytotoxic phytoalexins and transcriptional mediators. Proc. Natl. Acad. Sci. U.S.A. 112, 11407–11412. doi: 10.1073/pnas.1511131112 26305953 PMC4568653

[B17] Clifford J. C. Rapicavoli J. N. Roper M. C. (2013). A rhamnose-rich O-antigen mediates adhesion, virulence, and host colonization for the xylem-limited phytopathogen *Xylella fastidiosa* . Mol. Plant Microbe Interact. 26, 676–685. doi: 10.1094/MPMI-12-12-0283-R 23441576

[B18] Cruz S. S. Chapman A. G. Roberts I. M. Roberts D. A. Oparka K. J. (1996). Assembly and movement of a plant virus carrying a green fluorescent protein overcoat. Proc. Natl. Acad. Sci. U. S. A 93, 6286–6290. doi: 10.1073/pnas.93.13.6286 8692807 PMC39014

[B19] Dandekar A. M. Gouran H. Ibáñez A. M. Uratsu S. L. Agüero C. B. McFarland S. . (2012). An engineered innate immune defense protects grapevines from pierce disease. Proc. Natl. Acad. Sci. U. S. A 109, 3721–3725. doi: 10.1073/pnas.1116027109 22355130 PMC3309795

[B20] Dandekar A. M. Jacobson A. Ibáñez A. M. Gouran H. Dolan D. L. Agüero C. B. . (2019). Trans-graft protection against pierce’s disease mediated by transgenic grapevine rootstocks. Front. Plant Sci. 10. doi: 10.3389/fpls.2019.00084 PMC6372540 30787937

[B21] De La Fuente L. Parker J. K. Oliver J. E. Granger S. Brannen P. M. van Santen E. . (2013). The bacterial pathogen *Xylella fastidiosa* affects the leaf ionome of plant hosts during infection. PloS One 8, e62945. doi: 10.1371/journal.pone.0062945 23667547 PMC3646994

[B22] Dickmeis C. Honickel M. M. A. Fischer R. Commandeur U. (2015). Production of hybrid chimeric PVX particles using a combination of TMV and PVX-based expression vectors. Front. Bioeng. Biotechnol. 3. doi: 10.3389/fbioe.2015.00189 PMC4653303 26636076

[B23] Dongiovanni C. Di Carolo M. Fumarola G. Ciniero A. Tauro D. Palmisano F. . (2017). Recenti sperimentazioni per il controllo di xylella. Olivo e Olio 4, 25–29. doi: 10.5281/zenodo.833397

[B24] Donnelly M. L. Hughes L. E. Luke G. Mendoza H. Dam E. T. Gani D. . (2001). The ‘cleavage’ activities of foot-and-mouth disease virus 2A site-directed mutants and naturally occurring ‘2A-like’ sequences. J. Gen. Virol. 82, 1027. doi: 10.1099/0022-1317-82-5-1027 11297677

[B25] EFSA (2020). Update of the *Xylella* spp. host plant database - systematic literature search up to 30 June 2019. EFSA J. 18, 6114. doi: 10.2903/j.efsa.2020.6114 PMC7448098 32874307

[B26] EFSA PLH Panel Jeger M. Caffier D. Candresse T. Chatzivassiliou E. Dehnen-Schmutz K. . (2018). Scientific opinion on the updated pest categorization of *Xylella fastidiosa* . EFSA J. 16, 5357. doi: 10.2903/j.efsa.2018.5357

[B27] EFSA (2022). Update of the *Xylella* spp. host plant database - systematic literature search up to 31 December 2021. EFSA J. 20, 7356. doi: 10.2903/j.efsa.2022.7356 PMC9198695 35734284

[B28] EPPO (2006). PM 3/64 (1) intentional import of organisms that are plant pests or potential plant pests. EPPO Bull. 36, 191–194. doi: 10.1111/j.1365-2338.2006.00908.x

[B29] EPPO (2019). PM 7/24 (4) *Xylella fastidiosa* . EPPO Bull. 49, 175–227. doi: 10.1111/epp.12575

[B30] Francis M. Civerolo E. L. Bruening G. (2008). Improved bioassay of *Xylella fastidiosa* using *Nicotiana tabacum* cultivar SR1. Plant Dis. 92, 14–20. doi: 10.1094/PDIS-92-1-0014 30786389

[B31] Ingel B. Reyes C. Massonnet M. Boudreau B. Sun Y. Sun Q. . (2020). *Xylella fastidiosa* causes transcriptional shifts that precede tylose formation and starch depletion in xylem. Mol. Plant Pathol. 22, 175–188. doi: 10.1111/mpp.13016 33216451 PMC7814960

[B32] Jung Y.-Y. Kang K.-K. (2014). Application of antimicrobial peptides for disease control in plants. Plant Breed. Biotech. 2, 1–13. doi: 10.9787/PBB.2014.2.1.001

[B33] Kovalskaya N. Zhao Y. Hammond R. H. (2011). Antibacterial and antifungal activity of a snakin-defensin hybrid protein expressed in tobacco and potato plants. Open Plant Sci. J. 5, 29–42. doi: 10.2174/1874294701105010029

[B34] Kubaa R. A. Giampetruzzi A. Altamura G. Saponari M. Saldarelli P. (2019). Infections of the *Xylella fastidiosa* subsp. *pauca* strain "De donno" in alfalfa (*Medicago sativa*) elicits an overactive immune iesponse. Plants (Basel) 8, 335. doi: 10.3390/plants8090335 31500293 PMC6784145

[B35] Kyrkou I. Pusa T. Ellegaard-Jensen L. Sagot M.-F. Hansen L. H. (2018). Pierce’s disease of grapevines: a review of control strategies and an outline of an epidemiological model. Front. Microbiol. 9. doi: 10.3389/fmicb.2018.02141 PMC6143690 30258423

[B36] Laemmli U. K. (1970). Cleavage of structural proteins during the assembly of the head of bacteriophage T4. Nature 227, 680–685. doi: 10.1038/227680a0 5432063

[B37] Lee K. L. Uhde-Holzem K. Fischer R. Commandeur U. Steinmetz N. F. (2014). Genetic engineering and chemical conjugation of potato virus X. Methods Mol. Biol. 1108, 3–21. doi: 10.1007/978-1-62703-751-8_1 24243237 PMC5207041

[B38] Lico C. Benvenuto E. Baschieri S. (2015). The two-faced potato virus X: from plant pathogen to smart nanoparticle. Front. Plant Sci. 6. doi: 10.3389/fpls.2015.01009 PMC4646960 26635836

[B39] Livak K. J. Schmittgen T. D. (2001). Analysis of relative gene expression data using real-time quantitative PCR and the 2-ΔΔC_T_ method. Methods 25, 402–408. doi: 10.1006/meth.2001.1262 11846609

[B40] Lopes S. A. Ribeiro D. M. Roberto P. G. França S. C. Santos J. M. (2000). *Nicotiana tabacum* as an experimental host for the study of plant-*Xylella fastidiosa* interactions. Plant Dis. 84, 827–830. doi: 10.1094/PDIS.2000.84.8.827 30832132

[B41] López-García B. San Segundo B. Coca M. (2012). “Antimicrobial peptides as a promising alternative for plant disease protection,” in Small wonders: peptides for disease control. Eds. Rajasekaran K. Cary J. W. Jaynes J. M. Montesinos E. (Washington DC: American Chemical Society), 263–294.

[B42] Meyer M. M. Kirkpatrick B. C. (2011). Exogenous applications of abscisic acid increase curing of pierce's disease-affected grapevines growing in pots. Plant Dis. 95, 173–177. doi: 10.1094/PDIS-06-10-0446 30743407

[B43] Moll L. Baró A. Montesinos L. Badosa E. Bonaterra A. Montesinos E. (2022). Induction of defense responses and protection of almond plants against *Xylella fastidiosa* by endotherapy with a bifunctional peptide. Phytopathology 112, 1907–1916. doi: 10.1094/PHYTO-12-21-0525-R 35384723

[B44] Monteiro P. B. Renaudin J. Jagoueix-Eveillard S. Ayres A. J. Garnier M. Bové J. M. (2001). *Catharanthus roseus*, an experimental host plant for the *Citrus* strain of *Xylella fastidiosa* . Plant Dis. 85, 246–251. doi: 10.1094/PDIS.2001.85.3.246 30832036

[B45] Montesinos E. (2007). Antimicrobial peptides and plant disease control. FEMS Microbiol. Lett. 270, 1–11. doi: 10.1111/j.1574-6968.2007.00683.x 17371298

[B46] Montesinos E. Badosa E. Cabrefiga J. Planas M. Feliu L. Bardají E. (2012). “Antimicrobial peptides for plant disease control,” in Small wonders: peptides for disease control. Eds. Rajasekaran K. Cary J. W. Jaynes J. M. Montesinos E. (Washington DC: American Chemical Society), 235–261.

[B47] Montesinos L. Bundó M. Badosa E. San Segundo B. Coca M. Montesinos E. (2017). Production of BP178, a derivative of the synthetic antibacterial peptide BP100, in the rice seed endosperm. BMC Plant Biol. 17, 63. doi: 10.1186/s12870-017-1011-9 28292258 PMC5351061

[B48] Montesinos L. Gascón B. Ruz L. Badosa E. Planas M. Feliu L. . (2021). A bifunctional synthetic peptide with antimicrobial and plant elicitation properties that protect tomato plants from bacterial and fungal infections. Front. Plant Sci. 12. doi: 10.3389/fpls.2021.756357 PMC8558481 34733307

[B49] Moralejo E. Borràs D. Gomila M. Montesinos M. Adrover F. Juan A. . (2019). Insights into the epidemiology of pierce’s disease in vineyards of mallorca, Spain. Plant Pathol. 68, 1458–1471. doi: 10.1111/ppa.13076

[B50] Muranaka L. S. Giorgiano T. E. Takita M. A. Forim M. R. Silva L. F. Coletta-Filho H. D. . (2013). N-acetylcysteine in agriculture, a novel use for an old molecule: focus on controlling the plant-pathogen *Xylella fastidiosa* . PloS One 8, 8e72937. doi: 10.1371/journal.pone.0072937 PMC3751844 24009716

[B51] Nadal A. Montero M. Company N. Badosa E. Messeguer J. Montesinos L. . (2012). Constitutive expression of transgenes encoding derivatives of the synthetic antimicrobial peptide BP100: impact on rice host plant fitness. BMC Plant Biol. 12, 159. doi: 10.1186/1471-2229-12-159 22947243 PMC3514116

[B52] Navarro L. Jay F. Nomura K. He S. Y. Voinnet O. (2008). Suppression of the microRNA pathway by bacterial effector proteins. Science 321, 964–967. doi: 10.1126/science.1159505 18703740 PMC2570098

[B53] O'Donnell P. J. Jones J. B. Antoine F. R. Ciardi J. Klee H. J. (2001). Ethylene-dependent salicylic acid regulates an expanded cell death response to a plant pathogen. Plant J. 25, 315–323. doi: 10.1046/j.1365-313x.2001.00968.x 11208023

[B54] Oliveras A. Baró A. Montesinos L. Badosa E. Montesinos E. Feliu L. . (2018). Antimicrobial activity of linear lipopeptides derived from BP100 towards plant pathogens. PloS One 13, e0201571. doi: 10.1371/journal.pone.0201571 30052685 PMC6063448

[B55] Pagán I. García-Arenal F. (2020). Tolerance of plants to pathogens: a unifying view. Annu. Rev. Phytopathol. 58, 77–96. doi: 10.1146/annurev-phyto-010820-012749 32403981

[B56] Pereira W. E. L. Ferreira C. B. Caserta R. Melotto M. de Souza A. A. (2019). Xylella fastidiosa subsp. pauca and fastidiosa colonize arabidopsis systemically and induce anthocyanin accumulation in infected leaves. Phytopathology 109 (2), 225–232. doi: 10.1094/PHYTO-05-18-0155-FI 30277118

[B57] Pfaffl M. W. Horgan G. W. Dempfle L. (2002). Relative expression software tool (REST) for group-wise comparison and statistical analysis of relative expression results in real-time PCR. Nucleic Acids Res. 30, e36. doi: 10.1093/nar/30.9.e36 11972351 PMC113859

[B58] Randall J. J. Goldberg N. P. Kemp J. D. Radionenko M. French J. M. Olsen M. W. . (2009). Genetic analysis of a novel *Xylella fastidiosa* subspecies found in the southwestern united states. Appl. Environ. Microbiol. 75, 5631–5638. doi: 10.1128/AEM.00609-09 19581467 PMC2737921

[B59] Röder J. Dickmeis C. Fischer R. Commandeur U. (2018). Systemic infection of *Nicotiana benthamiana* with *Potato virus x* nanoparticles presenting a fluorescent iLOV polypeptide fused directly to the coat protein. Biomed. Res. Int. 2018, 9328671. doi: 10.1155/2018/9328671 29662905 PMC5831704

[B60] Rogers E. E. (2012). Evaluation of *Arabidopsis thaliana* as a model host for *Xylella fastidiosa* . Mol. Plant Microbe Interact. 25, 747–754. doi: 10.1094/MPMI-11-10-0270 22397407

[B61] Saitoh H. Kiba A. Nishihara M. Yamamura S. Suzuki K. Terauchi R. (2001). Production of antimicrobial defensin in *Nicotiana benthamiana* with a potato virus X vector. Mol. Plant Microbe Interact. 14, 111–115. doi: 10.1094/MPMI.2001.14.2.111 11204773

[B62] Shukla S. Dickmeis C. Nagarajan A. S. Fischer R. Commandeur U. Steinmetz N. F. (2014). Molecular farming of fluorescent virus-based nanoparticles for optical imaging in plants, human cells and mouse models. Biopamater. Sci. 2, 784. doi: 10.1039/C3BM60277J 32481848

[B63] Sindarovska Y. Kuchuk M. (2021). Long-term *Potato virus x* (PVX)-based transient expression of recombinant GFP protein in *Nicotiana benthamiana* culture *in vitro* . Plants 10, 2187. doi: 10.3390/plants10102187 34685995 PMC8537016

[B64] Singh H. Kaur K. Singh M. Kaur G. Singh P. (2020). Plant cyclophilins: multifaceted proteins with versatile roles. Front. Plant Sci. 11. doi: 10.3389/fpls.2020.585212 PMC7641896 33193535

[B65] Tollenaere C. Lacombe S. Wonni I. Barro M. Ndougonna C. Gnacko F. . (2017). Virus-bacteria rice co-infection in Africa: Field estimation, reciprocal effects, molecular mechanisms, and evolutionary implications. Front. Plant Sci. 8. doi: 10.3389/fpls.2017.00645 PMC5410622 28507553

[B66] Towbin H. Staehelin T. Gordon J. (1979). Electrophoretic transfer of proteins from poly- acrylamide gels to nitrocellulose sheets: Procedure and some applications. Proc. Natl. Acad. Sci. U. S. A 76, 4350–4354. doi: 10.1073/pnas.76.9.4350 388439 PMC411572

[B67] Ueno B. Funada C. K. Yorinori M. A. Leite R. P. Jr. (1998). First report of *Xylella fastidiosa* on *Catharanthus roseus* in Brazil. Plant Dis. 82, 712. doi: 10.1094/PDIS.1998.82.6.712A 30857035

[B68] van der Linde K. Hemetsberger C. Kastner C. Kaschani F. van der Hoorn R. A. Kumlehn J. . (2012a). A maize cystatin suppresses host immunity by inhibiting apoplastic cysteine proteases. Plant Cell 24, 285–1300. doi: 10.1105/tpc.111.093732 PMC3336116 22454455

[B69] van der Linde K. Mueller A. Hemetsberger C. Kaschani F. van der Hoorn R. A. Doehlemann G. (2012b). The maize cystatin CC9 interacts with apoplastic cysteine proteases. Plant Signal. Behav. 7, 1397–1401. doi: 10.4161/psb.21902 22960758 PMC3548856

[B70] Van Esse H. P. Reuber T. L. van der Does D. (2020). Genetic modification to improve disease resistance in crops. New Phytol. 225, 70–86. doi: 10.1111/nph.15967 31135961 PMC6916320

[B71] Visser M. Stephan D. Jaynes J. M. Burger J. T. (2012). A transient expression assay for the *in planta* efficacy screening of an antimicrobial peptide against grapevine bacterial pathogens. Lett. Appl. Microbiol. 54, 543–551. doi: 10.1111/j.1472-765X.2012.03244.x 22435990

[B72] Zhang S. Jain M. Fleites L. A. Rayside P. A. Gabriel D. W. (2019). Identification and characterization of menadione and benzethonium chloride as potential treatments of pierce's disease of grapevines. Phytopathology 109, 233–239. doi: 10.1094/PHYTO-07-18-0244-FI 30407880

[B73] Zvereva A. S. Golyaev V. Turco S. Gubaeva E. G. Rajeswaran R. Schepetilnikov M. V. . (2016). Viral protein suppresses oxidative burst and salicylic acid-dependent autophagy and facilitates bacterial growth on virus-infected plants. New Phytol. 211, 1020–1034. doi: 10.1111/nph.13967 27120694

