# Supplementary material for: Nicotiana benthamiana as a model plant host for Xylella fastidiosa: Control of infections by transient expression and endotherapy with a bifunctional peptide
Source: Front Plant Sci. 2022 Dec 1;13:1061463. doi: 10.3389/fpls.2022.1061463 (PMC9752042; doi:10.3389/fpls.2022.1061463)
Supplement: Supplementary file 1 [file Table_1.docx]

Supplementary Table 1. Genes and primers used for RT-qPCR analysis to quantify their expression levels in *Nicotiana* *benthamiana* plants

| **Gene** | **Primer code** | **Sequence (5'-3')** | **Concentration (nM)** | **Reference** |
| --- | --- | --- | --- | --- |
| *LRR receptor-like kinase, Flagellin signalling* | Nb BAK1 F  Nb BAK1 R | CTTATTCGGGTAGCTCTTCTCT  GTCTAACAAACACCCACTATCTGA | 200 | Chakravarthy et al., 2010 |
|  |  |  |  |  |
| *Cathepsin B protease, peptidase C1_Papain* | Nb Cath F  Nb Cath R | AGCGCCTTCTTGGAGTTA  TGGGATATGCAGGTTCACA | 200 | Chakravarthy et al., 2010 |
|  |  |  |  |  |
| *Cyclophilin, Protein folding* | Nb Cyc F  Nb Cyc R | CCTTACTATCGGCGGCACACCAG  GATCCAACAGCCTCAGCCTTCTTA | 200 | Chakravarthy et al., 2010 |
|  |  |  |  |  |
| *Cyclophilin, Protein folding* | Nb CycTC9299 F  Nb CycTC9299 R | GCAAGCCGTTACACTACAAAGGAT  CCCGAAAACCGACCCACAATAAG | 200 | Chakravarthy et al., 2010 |
|  |  |  |  |  |
| *Cytochrom C* | Nb CytC F  Nb CytC R | AATATGGCTGTGATGTGGGA  CAATGGTTTATCTTCCTGCG | 200 | Chakravarthy et al., 2010 |
|  |  |  |  |  |
| *Hydroxycinnamoyl/benzoyl-CoA* | Nb HCBT F  Nb HCBT R | TGCCATAGATGAGCCAAAAC  TCAATCGCCTTAAACCTCACTCTC | 200 | Chakravarthy et al., 2010 |
|  |  |  |  |  |
| *Plastocianin, Blue copper protein* | Nb Plasto F  Nb Plasto R | ATCCCATACAAGACGCTAAAACAA  GCTAACCCGCCTACACG | 200 | Chakravarthy et al., 2010 |
|  |  |  |  |  |
| *Lipoxygenase 9* | Nb-9-LOX F  Nb-9-LOX R | ATATGTGCCAAGGGACGA  AATAGGCCTTCGCCATCA | 200 | Huang and Schwab, 2011 |
|  |  |  |  |  |
| *PR-1, Pathogenesis related 1a* | PR1-1a F  PR1-1a R | CCTCGTACATTCTCATGGTCAAT  CCATTGTTACACTGAACCCTAGC | 200 | Wang et al., 2015 |
|  |  |  |  |  |
| *Plant defensine 1.2* | PDF1.2 F  PDF1.2 R | GGAAATGGCAAACTCCATGCG  ATCCTTCGGTCAGACAAACG | 200 | Wang et al., 2015 |
|  |  |  |  |  |
| *Phenylalanine ammonia-lyase* | PAL F  PAL R | GTTATGCTCTTAGAACGTCGCCC  CCGTGTAATGCCTTGTTTCTTGA | 200 | Wang et al., 2015 |
|  |  |  |  |  |
| *Ethylene responsive factor* | ERF1 F  ERF1 R | GGCGAATTTTCCGGGAGACT  GGCTCCGATTTTACTTCGCC | 200 | Abbas et al., 2018 |
|  |  |  |  |  |
| *PR-5, Thaumatin like protein* | PR5 F  PR5 R | CCGAGGTAATTGTGAGACTGGAG  CCTGATTGGGTTGATTAAGTGCA | 200 | Wang et al., 2015 |
|  |  |  |  |  |
| *Respiratory burst oxidase homolog* | RBOHB F  RBOHB R | TTTTCTCTGAGGTTTGCCAGCCACCA  GCCTTCATGTTGTTGACAATGTCTTT | 200 | Abbas et al., 2018 |
|  |  |  |  |  |
| *PR-2, β 1,3-Glucanase* | PR2 F  PR2 R | GGGCTGTTAATTTGCAGTATCC  GGTTTATAACATCTTGGTCTGATGG | 200 | Raffaello and Asiegbu, 2017 |
|  |  |  |  |  |
| *PR3, Endochitinase* | PR3 F  PR3 R | TGCCTTTTTCGGTCAAACTT  TGTAAATGGTTCTGCACTCAGG | 200 | Raffaello and Asiegbu, 2017 |
|  |  |  |  |  |
| *PR-4, Barwin domain chitinase* | PR4a F  PR4a R | CAACCCACAGAACATTAACTGG  TTGTCGGCATCCCAAGTAGT | 200 | Raffaello and Asiegbu, 2017 |
|  |  |  |  |  |
| *Endochitinase B* | Endo B F  Endo B R | GCCTTTATCAATGCTGCTAGG  ATCCTCGGGCAGTAGTATCG | 200 | Raffaello and Asiegbu, 2017 |
|  |  |  |  |  |
| *PR1, Pathogenesis related protein 1* | PR1 F  PR1 R | GGATGCCCATAACACAGCTC  GCTAGGTTTTCGCCGTATTG | 100 | Obrepalska-Steplowska et al., 2018 |
|  |  |  |  |  |
| *Phenylalanine ammonia lyase 2* | PAL2 F  PAL2 R | GATTGGAGCTTTCGAAGACG  CGGTGATCGGACTCTTTCTC | 100 | Yang et al., 2019 |
|  |  |  |  |  |
| *PR4, Pathogenesis related protein 4* | PR4 F  PR4 R | GGCCAAGATTCCTGTGGTAGAT  CACTGTTGTTTGAGTTCCTGTTCCT | 300 | Villarroel et al., 2016 |
|  |  |  |  |  |
| *Trypsin proteinase inhibitor* | TPI F  TPI R | ACTTTCGAATGCGATCCAAG  TCAACCACTTTGCTGCCATA | 100 | Villarroel et al., 2016 |
|  |  |  |  |  |
| *Mitogen-activated protein kinase 3* | MAPK3 F  MAPK3 R | GTCTGCTCGGTGTTGAATACGG  CCAATTACATTTTCATGGTCTAAATGGCG | 100 | López et al., 2020 |
|  |  |  |  |  |
| *WRKY transcriptional factor 22* | WRKY22 F  WRKY22 R | CCCGGAAACAAGTGGAGCGA  CTTCCGGCGAGAGAGTTCCG | 100 | Ramos et al., 2021 |
|  |  |  |  |  |
| *WRKY transcriptional factor 25* | WRKY25 F  WRKY25 R | CATGCGGTGGTCAGAAGCTG  CCGCTGGAACTAGTGGTGGT | 300 | Ramos et al., 2021 |
|  |  |  |  |  |
| *Actin* | Actin F  Actin R | GTGAAGGAGAAGTTGGCTTAC  CTTCTGGGCAGCGGAATCTC | 100 | Obrepalska-Steplowska et al., 2018 |
