# Supplementary figures and images for: Nicotiana benthamiana as a model plant host for Xylella fastidiosa: Control of infections by transient expression and endotherapy with a bifunctional peptide
Source: Front Plant Sci. 2022 Dec 1;13:1061463. doi: 10.3389/fpls.2022.1061463 (PMC9752042; doi:10.3389/fpls.2022.1061463)

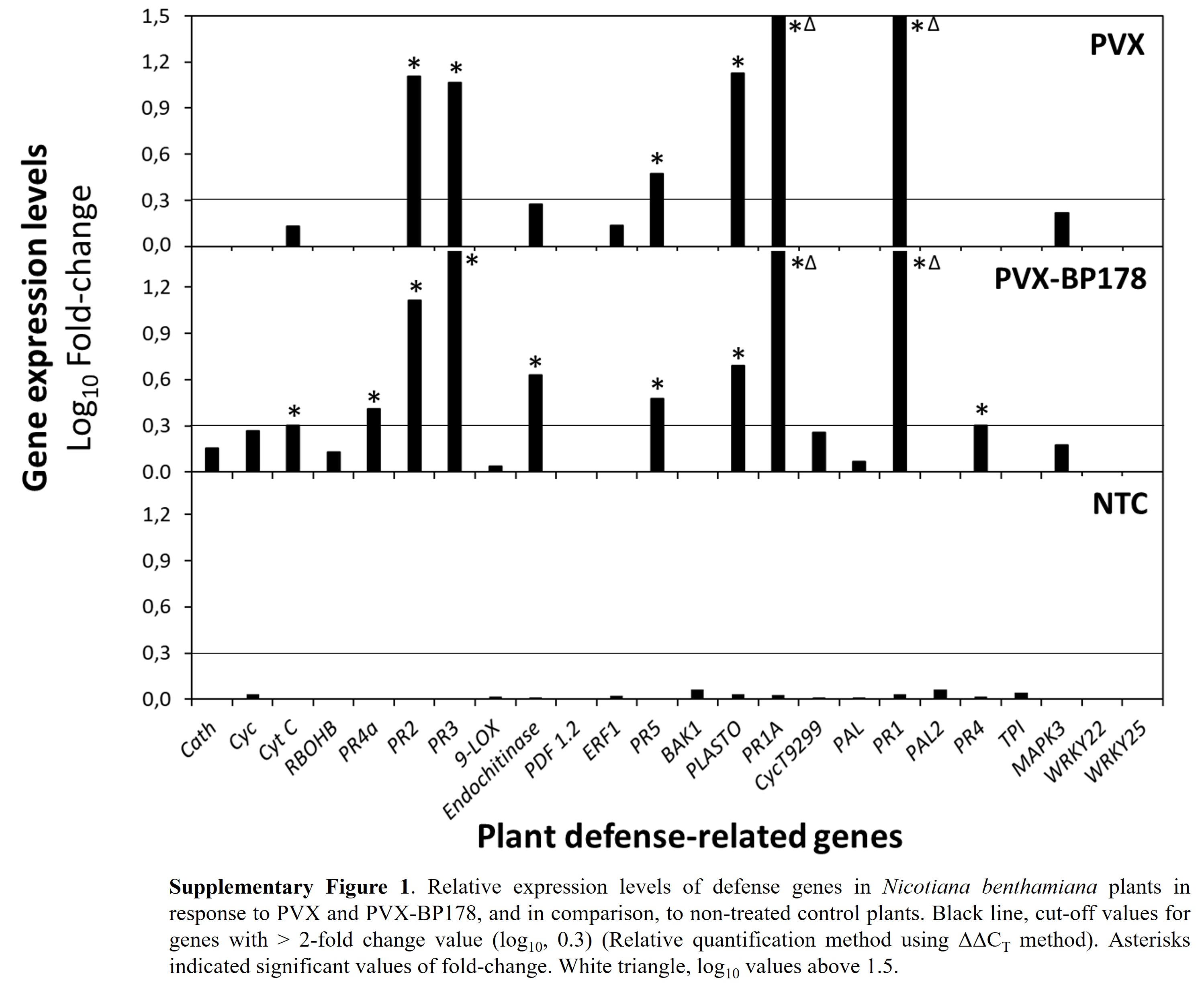

Supplement: Supplementary file 2 [file Image_1.tif]
